# Supplementary figures and images for: Association between genetic clades and cancer prevalence suggested by French-wide study of oncogenic small ruminant β-retrovirus diversity
Source: Front Cell Infect Microbiol. 2024 Nov 8;14:1466333. doi: 10.3389/fcimb.2024.1466333 (PMC11582038; doi:10.3389/fcimb.2024.1466333)

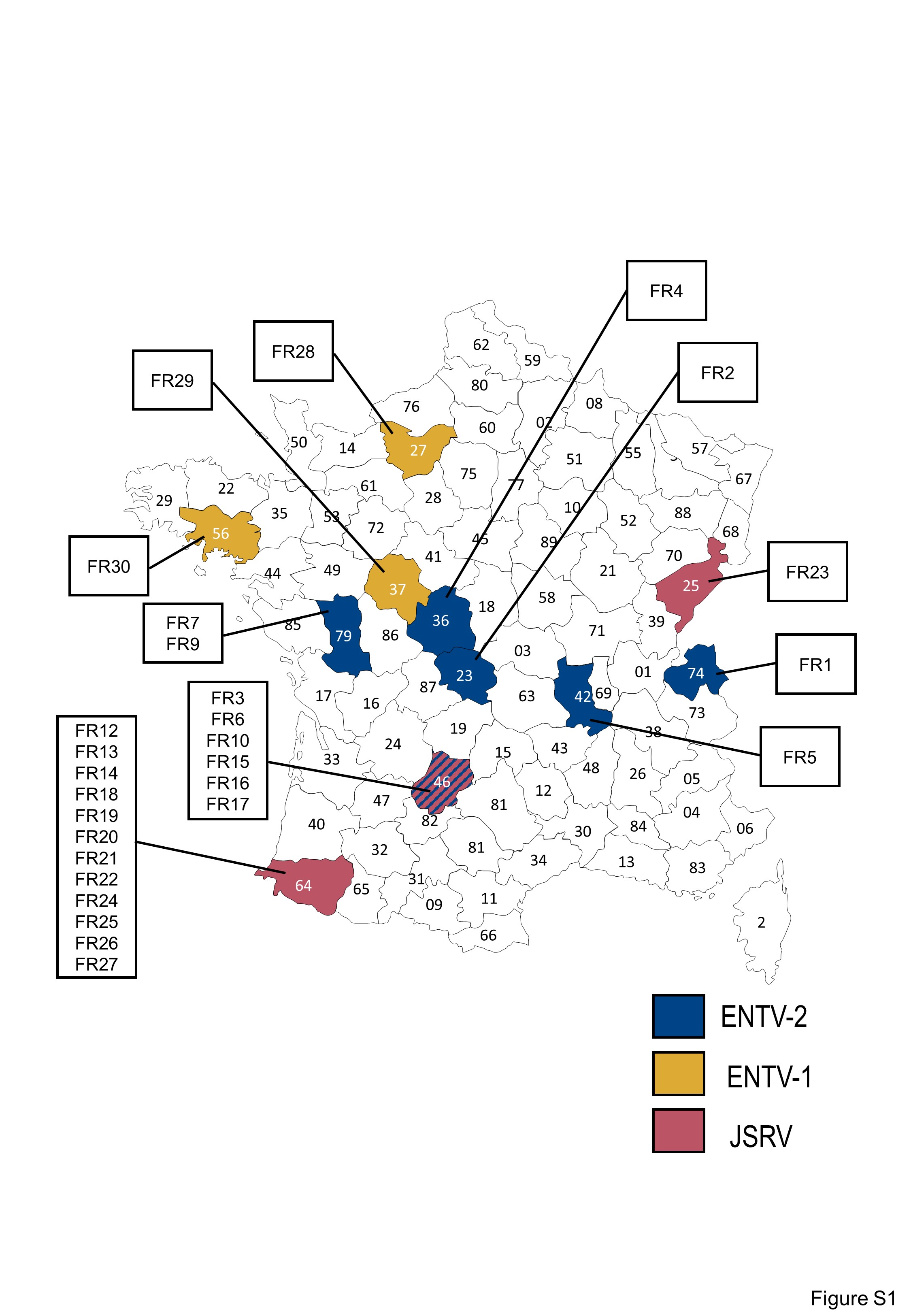

Supplement: Supplementary Figure 1 — Flock location on the French territory. The flocks from which JSRV and ENTV have been collected, are mapped according to their French department numbers. [file Image1.jpeg]

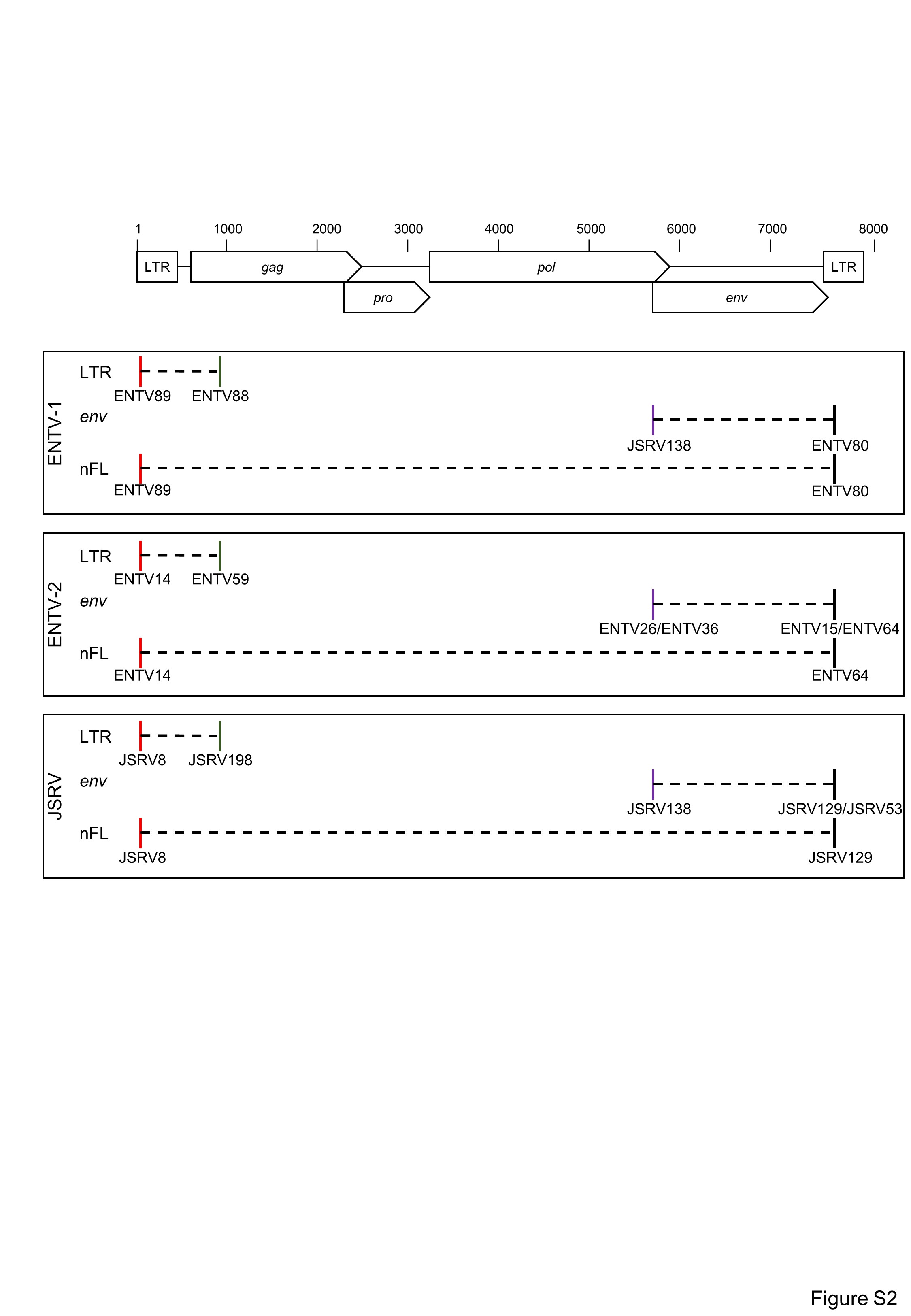

Supplement: Supplementary Figure 2 — Localization of PCR primers for the amplification of LTR, env and near full-length genome. For JSRV and ENTV-2 two different sets of primers were used for the amplification of env with the improvement throughout the study of the exo/endo amplification specificity. [file Image2.jpeg]

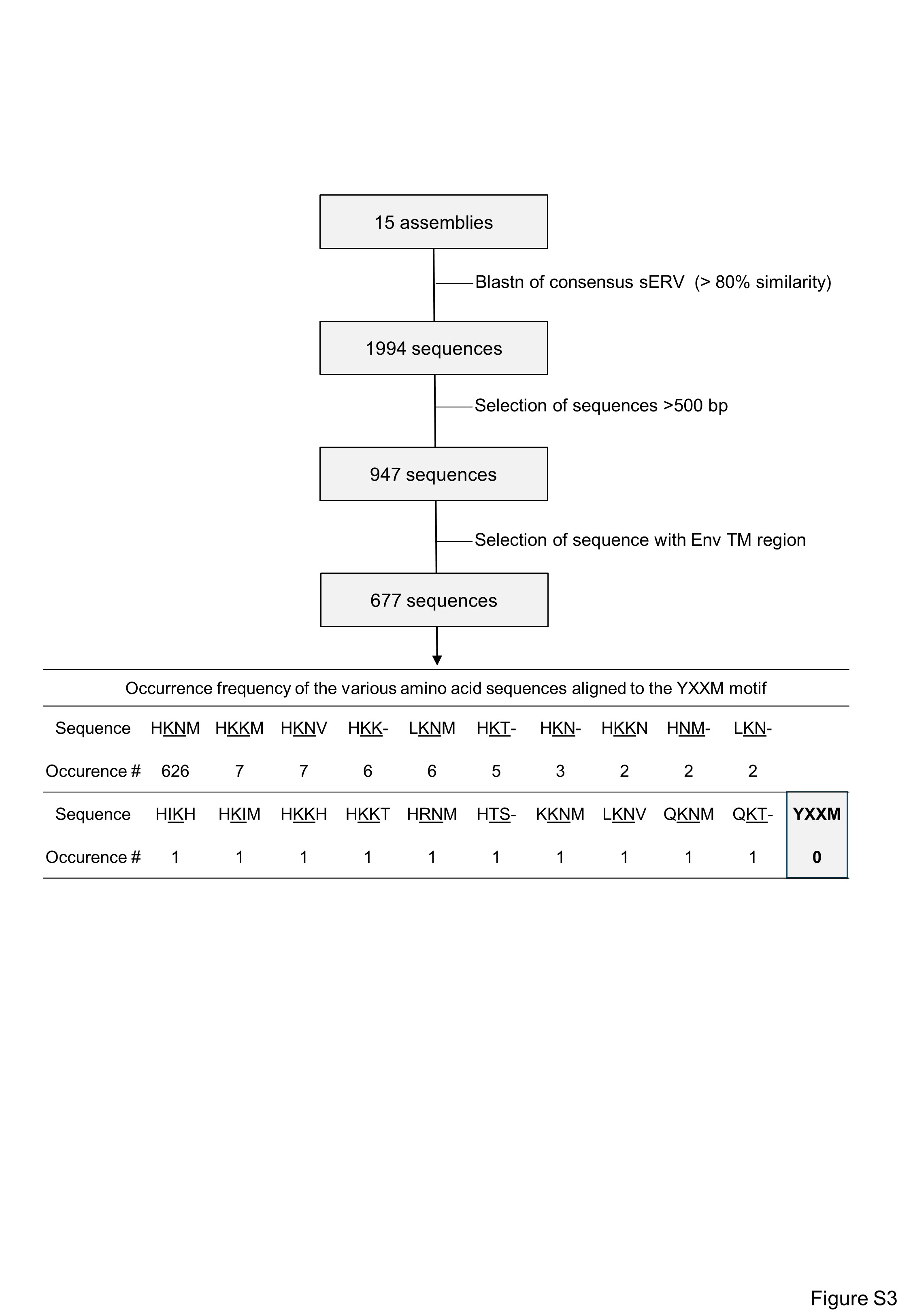

Supplement: Supplementary Figure 3 — Absence of the YXXM motif in the Env cytoplasmic tail of caprinae β-ERVs related to JSRV and ENTVs. sERV and gERV sequences within 15 caprinae assemblies (ARS1.2: GCA_001704415.1, ASM1117029: GCA_011170295.1, ASM1914517: GCA_019145175.1, ASM2665220: GCA_026652205.1, CapAeg_1.0: GCA_000978405.1, CAU_F1_maternal_1.0: GCA_023701675.1, CAU_O.aries_1.0: GCA_017524585.1, CVASU_BBG_1.0: GCA_004361675.1, Oar_v4.0: GCA_000298735.2, CAU_Oori_1.0: GCA_014523465.1, ARS-UI_Ramb_v2.0: GCA_016772045.1, Oar_ARS-UKY_Romanov_v1.0: GCA_022244705.1, Saanen_v1: GCA_015443085.1, Oar_ARS-UKY_WhiteDorper_v1.0: GCA_022244695.1, CHIR_2.0: GCA_000317765.2) were identified by BLASTn, using a consensus of sERV from sequences available in GenBank (see Figure 1 and Materials and methods). Sequences < 500 bp corresponding to solo LTRs were not included. MAFFT alignment of the different sequences allowed to select only sequences with a preserved TM region of Env. [file Image3.jpeg]

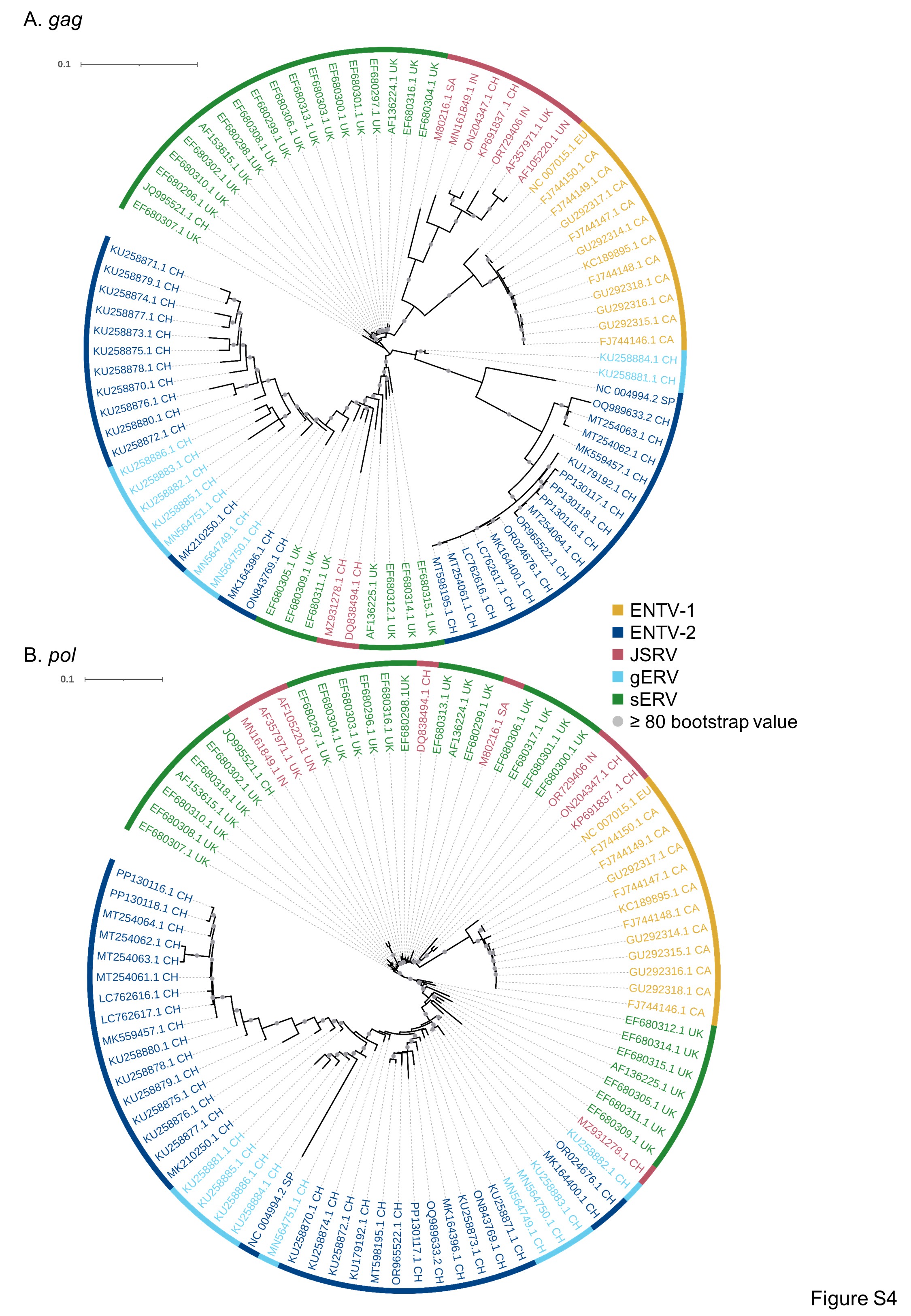

Supplement: Supplementary Figure 4 — Phylogenetic analysis of sequences of ENTV-1, ENTV-2 and JSRV and their related endogenous sequences from sheep and goats. Maximum likelihood tree with 10,000 ultrafast bootstrap replicated with EF680307.1 (enJSRV10) as root sequence for (A) gag (B) pol region. The scale bar indicates the nucleotide substitution per site. CH: China, UK: United Kingdom, CA: Canada, IN: India, SA: South Africa, SP: Spain, EU: Europe, PA: Pakistan, UN: Unknown. [file Image4.jpeg]

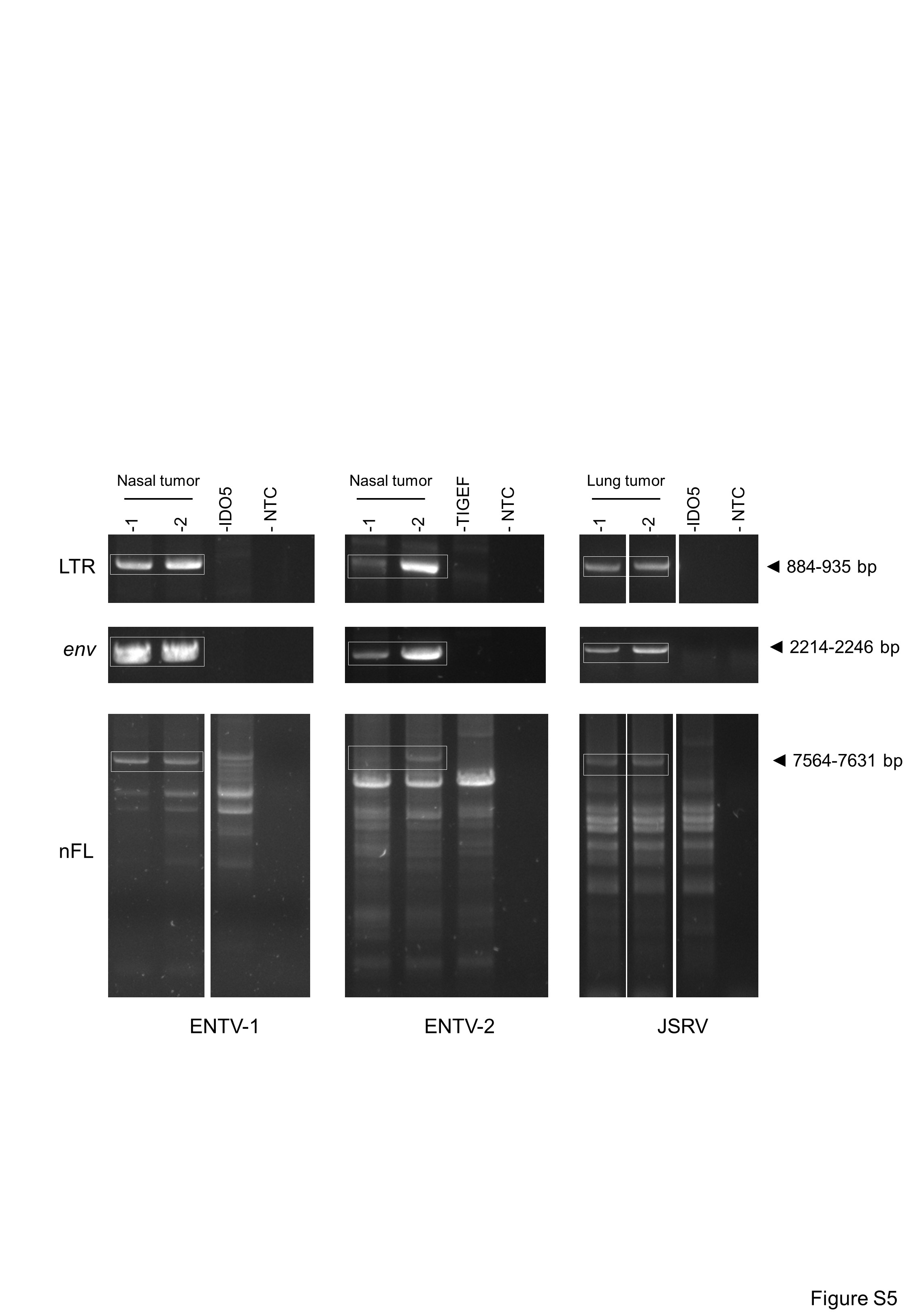

Supplement: Supplementary Figure 5 — Migration profile of ENTV and JSRV PCR amplification. For each PCR, the example of genomic DNA extracted from two tumors used as a template per virus is shown, in addition to DNA extracted from ovine (IDO5) and caprine (TIGEF) cell lines used as a control for endogenous amplification. nFL = near full-length. [file Image5.jpeg]
